# 2020 The clinical implications of a positive prostate cancer screen in patients undergoing a cardiac transplant evaluation

**DOI:** 10.1017/cts.2018.186

**Published:** 2018-11-21

**Authors:** Vaibhav Kumar, Hanyin Wang, David DeNofrio, David Kent

**Affiliations:** 1 Tufts University; 2 Tufts Medical Center

## Abstract

OBJECTIVES/SPECIFIC AIMS: Screening the general population for prostate cancer with prostate specific antigen (PSA) continues to be controversial. Patients with advanced heart failure undergoing evaluation for suitability for cardiac transplantation are often requested to undergo prostate cancer screening, with guiding evidence generated from the general population. The objective of this study is to determine the clinical implications of a positive prostate cancer screen result in this patient population. METHODS/STUDY POPULATION: A retrospective cohort study was performed on all men that were referred to a tertiary care cardiac transplant center between January 2000 and December 2015. Patients were classified as having either a “positive screen” (PSA≥4 ng/mL) or a “negative screen” (PSA<4 ng/mL) at the point of evaluation. The primary outcome of time to listing for cardiac transplant (days) was calculated from the date of referral to the date of listing. A multivariable Cox proportional hazards model was developed to assess the association between a positive prostate cancer test result and listing for cardiac transplantation. RESULTS/ANTICIPATED RESULTS: Among the 704 patients included in this study, 66 men (9.4%) had a positive prostate cancer screen result. Men with a positive prostate cancer screen were approximately 4 year older (mean 58.5 vs. 54.1 years), more likely to have a diagnosis of Ischemic Cardiomyopathy (74% vs. 53%) and require continuous mechanical support (61% vs. 16%) at the point of transplant evaluation. The median time for listing for cardiac transplant was greater in patients with a positive PSA (119 vs. 48 days, *p*<0.05). After adjusting for age, renal function, clinical status at evaluation, history of COPD, and year of referral, patients with a positive prostate cancer screen had a reduced hazards ratio (HR) for progressing to cardiac transplant listing compared with those with a negative screen (HR 0.58, 95%CI: 0.38–0.91). DISCUSSION/SIGNIFICANCE OF IMPACT: Screening patients undergoing cardiac transplant evaluation for prostate cancer with PSA has a low diagnostic yield. An individual’s PSA value is influenced by their age and clinical status at the time of screening, with a positive screen being associated with a reduced likelihood for progressing to listing for cardiac transplant.

